# Active School-Based Interventions to Interrupt Prolonged Sitting Improve Daily Physical Activity: A Systematic Review and Meta-Analysis

**DOI:** 10.3390/ijerph192215409

**Published:** 2022-11-21

**Authors:** Marta Amor-Barbosa, Anna Ortega-Martínez, Andoni Carrasco-Uribarren, Maria Caridad Bagur-Calafat

**Affiliations:** 1 Physiotherapy Department, Universitat Internacional de Catalunya, 08195 Barcelona, Spain; 2 Physiotherapy Department, Fundació Aspace Catalunya, 08001 Barcelona, Spain

**Keywords:** sitting interruption, daily physical activity, sedentary behavior, moderate-to-vigorous physical activity, school, children, adolescents

## Abstract

Background: Sedentary behavior (SB) is a negative routine for health, especially during advancing age. Promoting an active lifestyle and reducing SB is a global endeavor. The aim of this study was to analyze the effects of active school-based programs to interrupt prolonged sitting for daily physical activity (PA) and daily SB in children and adolescents. Methods: A systematic review was conducted. Clinical trials analyzing the effect of interrupting prolonged sitting with active breaks and classroom-based PA were included. Studies that implemented PA in class without interrupting prolonged sitting or those that implemented multimodal interventions were excluded. A systematic search was conducted in 6 databases: Medline, WOS, Cochrane Library, SPORTDiscus, CINAHL and EMBASE. Primary outcomes were daily PA and daily SB, while moderate-to vigorous physical activity (MVPA) was considered a secondary outcome. Results: Six studies were included, with a total of 976 participants between 6–14 years. The interventions were heterogeneous in duration of the sitting time interruption (5–30 min), frequency (1–3 times per-day up to three times per-week) and total duration (five days to three years). 50% of the studies scored “high risk” of bias. Three meta-analyses were performed for daily PA, MVPA and SB, showing a significant improvement in the daily PA and MVPA. Conclusions: School-based programs aimed to interrupt prolonged sitting could be a good strategy to improve daily PA and MVPA levels. (Registration number: CRD42022358933).

## 1. Introduction

Sedentary behavior (SB) is defined as “any waking behavior characterized by an energy expenditure ≤ 1.5 metabolic equivalents (METs), while in a sitting, reclining or lying posture” [1]. It has important implications in children’s health, as evidence suggests that SB may be related to low fitness and poor cardiometabolic health [2]. Some of the SB identified in children and youth from 5 to 17 years old include sitting at school and doing homework while sitting [1], but the most frequent SB in leisure time occurs during screen-based activities, such as watching TV or playing video games where children remain in a sitting or lying position for long periods of time [3,4]. SB guidelines recommend that children under four years should not spend more than one hour in screen-based activities [5], whereas for school-aged children and adolescents the recommendation increases to a maximum of two hours [6]. Despite this, the fact is that children and adolescents spend over three to four hours in SB, which is in line with research showing that SB increases with increasing age [7].

It is widely known that physical activity (PA) has multiple benefits in children and adolescents’ health conditions [8]. These include physical effects (e.g., cardiorespiratory and muscular fitness, bone health, obesity reduction, etc.), mental effects (e.g., reduction of depression and anxiety), and cognitive effects (e.g., school performance, memory, executive function, and attention, etc.) [9,10].

The World Health Organization (WHO) recommends a minimum of 60 min of moderate-to vigorous physical activity (MVPA) per day, as well as a reduction in the time spent in SB [2,8]. Notwithstanding the previously mentioned benefits of PA in health during childhood and adolescence, recent evidence indicates that trends in PA of children and adolescents have decreased in recent years [11], leading to three out of four adolescents from 11 to 17 years not accomplishing the recommendations [12]. This is in line with the statement that adolescents are prone to choose sedentary activities in detriment of active requirements [7].

The WHO’s action plan 2018–2030 recognized the school environment as a key setting for implementing PA in children and adolescents and established as a specific action for all-globe policies to promote and increase opportunities for PA in such environments [12]. Since then, different studies have been designed to increase active time and reduce sedentary time at school, with diverse approaches. However, methodologies and interventions vary among studies [13,14]. Some use static standing interventions, where standing desks are used during school-time, changing the way children attend the usual lessons. Others offer multimodal interventions, where a larger approach is utilized. They combine health recommendations, nutritional recommendations, educational programs and PA programs—among others—for children and families, and they are often related to governmental or global programs. The third type of interventions observed are PA programs performed during school time, in which bouts of PA are usually introduced in the school routine for breaking up prolonged sitting occurring during classroom time.

Previous systematic reviews [13,14] summarize the results of interventions on PA levels in any time slot of the day. To determine the degree of compliance with WHO recommendations, it is necessary to isolate studies in which the effects of interventions on PA levels are recorded throughout the entire day, excluding studies that measure the effect only during the active break or during school hours.

This review aims to analyze the effects of active school-based programs to interrupt prolonged sitting in daily physical activity and daily sedentary behavior in children and adolescents. In addition, it has been proposed as a secondary objective to analyze the effect of this intervention on daily moderate-to-vigorous physical activity.

## 2. Materials and Methods

### 2.1. Study Design

The protocol of the systematic review was registered in the International Prospective Register of Systematic Reviews (PROSPERO), with registration number CRD42022358933. The recommendations of the Preferred Reporting Items for Systematic Reviews and Meta-Analysis (PRISMA) statement and the Cochrane recommendations have been followed for this systematic review [15].

### 2.2. Search Strategy

Studies were identified by computer-assisted electronic searches of Medline, Web of Science, Cochrane Library, SPORTDiscus, CINAHL and EMBASE. The electronic search was performed with an ending date of December 2021. In addition, reference lists were screened manually to find other potentially relevant references.

The searching strategy included terms related to the population (“pediatrics” OR “children” OR “child” OR “toddler” OR “adolescent”), the intervention (“break” OR “break up” OR “breaking up” OR “bout” OR “interrupt”), and the comparison—defined as behavior—(“risk reduction behavior” OR “sedentary behavior” OR “sitting position” OR “posture” OR “rest” OR “prolonged sitting”), connected with Boolean operators adapted to each database. The search strategies in each database are available in the Supplementary Materials.

### 2.3. Criteria for Selection

For this review, only clinical trials were included (randomized clinical trials, clinical controlled trials, and cross-over studies). Interventions were only included if based on interrupting prolonged sitting with classroom-based PA. That means studies were excluded if interventions were: (a) static; meaning any intervention that did not include movement (e.g., static interventions using standing desks); (b) multimodal; meaning interventions including other activities apart from physical activity (e.g., nutritional recommendations, educational programs for families); (c) not completely performed during school time; (d) not completely performed at school. A duration of the intervention of more than one day was required to be included in this review. For the control group, the inclusion criteria was not receiving any specific intervention.

Studies were excluded if the sample included children and adolescents with disabilities who were not able to follow the intervention as set at the beginning. Finally, studies were excluded if daily PA and/or SB were not assessed as outcome measures or if they were only assessed during school hours; that is, not assessed during the whole day.

Types of instruments of measure included questionnaires, activity trackers, pedometers or triaxial accelerometers. Data regarding changes in daily PA levels in children and adolescents were collected from the baseline, post-intervention and follow-up measurements, if they were available. No restrictions were applied regarding publication year nor language.

### 2.4. Data Extraction

A division in two blocks of titles and abstracts found by the searching strategy was made. Two authors screened independently the first block of references (M.A.-B. and M.C.B.-C.), while the two other authors did the same with the second block (A.C.-U. and M.C.B.-C.), following the inclusion criteria described above. A.C.-U. acted as arbitrator for M.A.-B. and M.C.B.-C., while M.A.-B. fulfilled this role for A.C.-U. and M.C.B.-C. in the case of discrepancies. Selected titles were read in full-text to determine whether they were relevant to the review or not. In case of disagreement on the final eligibility, a decision-making process was done through discussion. Reasons for excluding trials were recorded.

The data analyzed in the review were extracted from text and tables given in the studies. When no access to data was possible, we contacted the authors. The extracted information included: (1) characteristics of the study population, (2) aspects of performed intervention (3) outcome measures, and (4) results. The data analysis was done using a qualitative synthesis and, when possible, a quantitative synthesis (meta-analysis).

### 2.5. Risk of Bias and Methodological Quality of Clinical Trials 

Two authors independently assessed both methodological quality and risk of bias of the clinical trials included in the review by means of the PEDro scale [16], and the Cochrane Risk of Bias tool [17]. M.C.B.-C. and M.A.-B. assessed the clinical trials methodological quality, while A.C.-U. and A.O.-M. assessed the potential risk of bias. Discrepancies were resolved throughout a consensus meeting, such as in the screening process of references. A.C.-U. and M.A.-B. acted as arbiters for both assessments, respectively.

The PEDro scale comprises 11 items. A score of 1 was awarded each time a criterion was satisfied, and a total score was obtained by summing up the results obtained from items 2 to 11. The potential risk of bias was assessed using the Cochrane Risk of Bias tool that classifies the studies as “low risk”, “unclear risk”, or “high risk” based on 6 criteria [17]. The PEDro scale is a reliable tool for evaluating the quality of the studies and assessing the risk of bias [18].

### 2.6. Data Synthesis and Analysis 

In Table 1, a descriptive synthesis of the included articles has been provided, including year of publication, country, age (age range and/or mean age), sample size, sitting time interruption, measured construct (PA, MVPA and/or SB), tool, and other outcomes.

Three different meta-analyses were performed for daily PA, MVPA, and SB. The meta-analyses were performed with the mean difference (MD) in final scores and standard deviations (SDs). Standardized mean differences (SMD) and 95% confidence intervals were calculated based on the data on the post-intervention means and SDs.

The *p* value significance was set at <0.05. Heterogeneity between the studies was assessed using both ✗^2^ test and I^2^ statistic. If I^2^ value was less than 50%, no heterogeneity was presumed, and fixed effect models were used, while when I^2^ was greater than 50%, mixed effects models were used. Meta-analyses were performed using RevMan 5.4. software (The Cochrane Collaboration, London, UK, 2020).

## 3. Results

### 3.1. Literature Search and Screening

**Figure 1:**
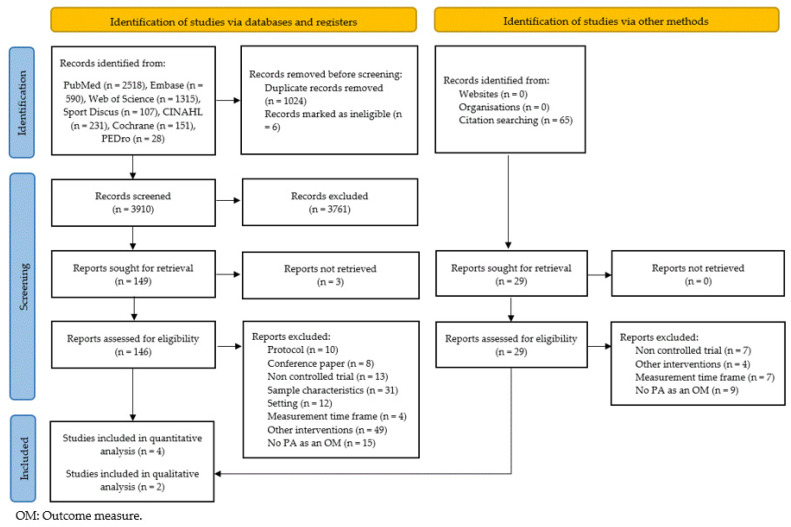
The flow diagram.

The initial search identified 3910 references. A manual search from reference lists of relevant articles was conducted for 65 other potentially relevant references (Figure 1). After exclusion by title and abstract, 175 articles were eligible for full-text screening. From these, 167 were excluded, being the most common reason for exclusion that the intervention did not aim at reducing sitting time at school (53 studies). Finally, 6 studies [19,20,21,22,23,24] were included in this review: 4 for quantitative analysis [19,20,21,22] and 2 for qualitative analysis [23,24]. The included studies were published between 2009 and 2020.

### 3.2. Characteristics of Eligible Studies

Table 1 shows the characteristics of the included studies classified by year of publication, country, age (age range and/or mean age), sample size, sitting time interruption, measured construct (PA, MVPA and/or SB), tool, and other outcomes. 

The studies included in this review recruited a total of 976 participants between 6 and 14 years. Only studies with samples of children aged 6–12 years were included in the quantitative synthesis [19,20,21,22]. Reported outcomes were daily PA (n = 3) [19,21,22], daily MVPA (n = 4) [19,20,21,22] and daily SB (n = 2) [21,22]. The tools used to measure PA were accelerometers (n = 3) [19,20,22], activity trackers (n = 1) [21] and questionnaires (n = 2) [23,24]. The interventions were heterogeneous in duration of sitting time interruption (between 5–30 min), frequency (from 1–3 times per day up to three times per week) and total duration of the intervention (from 5 days to 3 years). We only included studies with an intervention period between 4 weeks and 3 years in the meta-analyses [19,20,21,22]. The studies were conducted in the USA (n = 3) [19,21,24], Ireland (n = 1) [20], Italy (n = 1) [22] and Spain (n = 1) [23].

### 3.3. Risk of Bias and Methodological Quality of Clinical Trials

The methodological quality assessment of all the included studies was carried out following the PEDro scale, with the items listed in Table 2. The final mean score of quality was 4.2 points, ranging between 2 and 6. The most frequent recurring biases were related to the blinding, with only two studies masking the outcome assessor. Any study concealed the allocation. All papers reported “Point and variability measures” data and “Between-group statistical comparisons”, but no study applied the intention to treat analysis.

The punctuation obtained for each article in the Cochrane risk-of-bias assessment is included on the right side of each figure of meta-analysis. As shown, all the included studies scored “unclear risk” or “high risk” in the risk of bias assessment; that is, 25% and 75%, respectively. The most frequent risk of bias was related to the randomization process and the missing data, as most of the studies did not show baseline data and/or had losses or incomplete follow-ups. 50% of the studies scored “high risk” of bias in those items.

### 3.4. Synthesis of Results

- Daily PA

**Figure 2:**
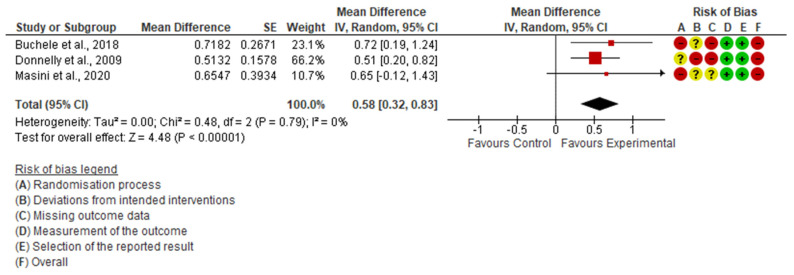
Forest Plot of daily PA [19,21,22].

Daily PA was measured in five studies; three were included in the quantitative analysis [19,21,22]. The meta-analysis showed that interrupting the prolonged sitting produced a significant improvement in daily PA (SMD = 0.58; 95% CI: 0.33, 0.83; I^2^: 0%) (Figure 2). 

Two studies were included in the qualitative synthesis. Pinto–Escalona et al. [23], showed statistically significant results in favor of the experimental group in frequency of PA compared to the control group (*p* = 0.000). In the study of Reed et al. [24], both experimental and control groups showed an improvement in the Previous Day Physical Activity Recall questionnaire. However, no differences were observed in the between-group analysis. None of the two studies could be included in the meta-analysis. They evaluated the effects of the intervention on PA with questionnaires [23,24].

- Daily SB

**Figure 3:**
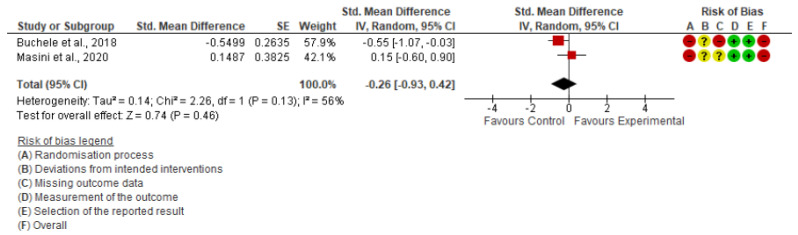
Forest Plot of daily SB [21,22].

The two included studies [21,22] in the meta-analysis were the only ones that reported daily SB. The quantitative analysis showed that interrupting prolonged sitting was not enough to produce a significant reduction in daily SB (SMD = −0.26; 95% CI: −0.93, 0.42; I^2^: 56%) (Figure 3).

- Daily MVPA

**Figure 4:**
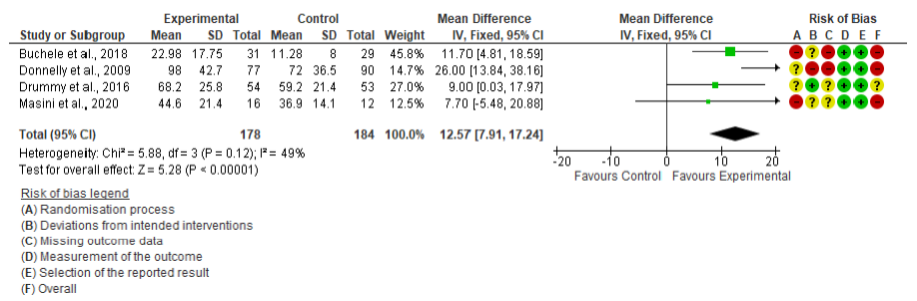
Forest Plot of daily MVPA [19,20,21,22].

Daily MVPA was measured in four of the included studies [19,20,21,22]. Meta-analysis showed that interrupting prolonged sitting in school produced a significant improvement in daily MVPA in school-aged children and adolescents (MD = 12.57; 95% CI: 7.91, 17.24; I^2^: 49%; SMD = 0.58; 95% CI: 0.37, 0.79; I^2^: 0%) (Figure 4).

## 4. Discussion

This systematic review and meta-analysis assessed the effect of different exercise-based interventions to interrupt prolonged sitting during school hours and their influence on PA, MVPA and SB levels throughout the day. Only six studies met the defined eligibility criteria. The meta-analyses showed that active school-based interventions for interrupting prolonged sitting increase levels of PA and MVPA performed throughout the day. Moreover, they may reduce the amount of time spent in SB, but no significant differences were found for SB reduction.

The review from Masini et al. [25] included studies that reported effects only on PA levels at school and during the entire day, but they did not differentiate the analysis of these effects. Comparing the two meta-analyses, Masini et al. [25] did not find statistically significant differences for the MVPA in favor of the intervention group, in contrast with ours, which did find them.

The review of Watson et al. [26] aimed at evaluating the impact of classroom-based PA interventions on academic-related outcomes. Its secondary objective was to examine the effect of these interventions on children’s PA levels. The results from the three studies included in the meta-analysis [27,28,29] indicated that classroom-based PA did not modify PA levels. In contrast, our meta-analysis showed that interrupting prolonged sitting produced a significant improvement in total daily PA.

The review conducted by Hegarty et al. [13] concluded that multi-component interventions, which also include the use of standing desks, may be effective methods for reducing children’s sitting time in a school-based intervention. The Cochrane Systematic Review from Neil-Sztramko et al. [14], as well as the one by the Hegarty et al. [13], showed that multi-component interventions were the most common interventions performed in a school environment. The results of our review are not in line with the two previous reviews cited, where it is observed that interventions that are solely based on PA interventions in the classroom are not enough to change the SB in children and adolescents. To our knowledge, this is the first Systematic Review that specifically analyses the effects of school-based active programs on daily levels of the three main components of the WHO recommendations—PA, MVPA and SB [2,12]. So, our Systematic Review contributes to completing the information given by older Systematic Reviews [13,14,25,26].

Although there are no established definitions for classroom-based PA, SB interruptions may include (a) short bouts of PA performed as a break from academic content (active breaks), (b) short bouts of PA including academic content (curriculum-focused active breaks), or (c) PA integrated into lessons in key learning areas, such as mathematics (physically active lessons). Three of the included studies in our review performed active breaks without academic content [20,21,22]. Only Pinto–Escalona et al. [23] included curriculum-focused active breaks. Two of the included studies performed physically active lessons [19,24].

Regarding the dosage of the interventions, heterogeneity in the duration of the sitting time interruption (bouts between 5–10 min), frequency (1–3 times per day up to three times per week) and total duration of the intervention (5 days to 3 years), was found. Differences in classroom-based PA interventions to interrupt sitting time and exercise dosage may account for the heterogeneity found in the meta-analyses.

From the six studies included in this review, two of them were not included in the quantitative synthesis [23,24]. Although Pinto–Escalona et al. [23] measured PA using the Questionnaire for Assessing Physical Activity in Teenage School Children, they did not provide data. Reed et al. [24] only reported the mean of PA (not the standard deviation) of the experimental group. Neither the baseline nor the post-intervention PA levels of the control group were reported. Participants in the experimental group were also given a pedometer (DIGI-WALKER, Yamax Inc., Tokyo, Japan) to record the steps immediately following the lesson, but these data were not treated as an outcome measure [24].

One important characteristic of the studies included in this Systematic Review is the way that outcomes are assessed. From different tools available for measuring PA, MVPA and SB, studies included used accelerometers (n = 3) [19,20,22], activity trackers (n = 1) [21] and questionnaires (n = 2) [23,24]. The meta-analysis did not include studies that recorded PA with questionnaires, for the reasons explained above. The study from Buchele et al. [21] was the only one to measure PA with an activity tracker.

The accelerometry data collection procedure differed among the included studies regarding time frame and data processing [19,20,22]. There is no clear consensus on the optimal time frame, but some authors recommend a monitoring period of at least six days between 8 and 10 h, although at least 10 h is preferable [30,31]. There are also no clear recommendations on data processing procedures in samples of participants between 6 and 18 years. It is suggested to use epochs lower than 15 s to detect the intermittent activity characteristic of children [31], but different algorithms to discriminate PA by intensity levels have been described [32,33].

Daily PA levels were measured in five studies, three of them [19,21,22] being included in the meta-analysis. They showed that interrupting prolonged sitting produced a significant improvement in daily PA. Studies included in the qualitative analysis [23,24] also showed positive effects after implementing active school-based programs to interrupt prolonged sitting. These positive results are in contradiction, as previously mentioned, with those of Watson et al. [26], that showed classroom-based PA did not affect PA level.

Chaput et al. [2], in their summary of 2020 WHO guidelines on physical activity and sedentary behavior for children and adolescents aged 5–17 years, highlighted that “the evidence reviewed on the associations between PA and health outcomes in children and adolescents reaffirms the findings reported in 2010” [8]. Nevertheless, the WHO introduced a change in their recommendations, from children doing at least 60 min of PA per day to doing at least 60 min of MVPA. Related to this change in the recommendation, current studies that aim at implementing strategies to promote PA focus specifically on MVPA, instead of PA in general.

Regarding the MVPA levels in school-aged children, our meta-analysis showed that the active interruption of SB at school is an efficient way to increase them, as previously found by Masini et al. [25]. Of all the studies included in the meta-analyses, those of Donnelly et al. [19] and Drummy et al. [20] found significant differences (*p* < 0.05) between the control and the experimental groups for daily MVPA. This is not surprising, considering that their interventions specifically introduced MVPA activities. Contrarily, those of Buchele et al. [21] and Masini et al. [22] did not show any significant differences between groups (*p* > 0.05), even though MVPA values were considerably higher after the intervention for the experimental groups, and between groups.

Tassitano et al. [34] previously mentioned that children should accomplish approximately 30 min of MVPA during school attendance. In fact, they demonstrated that this is usually achieved (or nearly) without any intervention needed, with an estimation of ~27.8 min/day MVPA. This goes in line with the baseline data presented by Drummy et al. (Experimental Group (EG) mean (M) = 58.7, SD = 16.8; Control Group (CG) M = 59.7, SD = 17.5), and Masini et al., (EG M = 36.5, SD = 18.4; CG M = 42.2, SD = 19.7), and CG data at follow-up showed by both Donnelly et al. (CG M = 72, SD = 36.5) and Drummy et al. (M = 59.2, SD = 21.4) [19,20,22]. 

Considering the WHO recommendations regarding MVPA—approximately 60 min per day—some comments can be made. Neither the studies by Buchele et al. nor Masini et al. achieved reaching the WHO recommendations, even at the end of their interventions [21,22]. This suggests that, even though school is a suitable environment for promoting PA [12], the recommended levels of MVPA may be accomplished by active routines that involve more than school time, or with programs that involve not only PA, but also nutritional education, among others.

About SB, given the wide range of cardiometabolic risk factors associated with SB (including HDL cholesterol, blood pressure, adiposity, and combined cardiometabolic risk), replacing prolonged sitting behavior with PA should be a global endeavor [35]. The school environment could be a very conducive place to promote PA in children [36], and it would be expected that the sedentary habit would be reduced if children remained more active during the school day [13]. In contrast, this meta-analysis showed that interrupting prolonged sitting with active school-based interventions is not enough to improve daily SB in children (SMD = −0.26; 95% CI: −0.93, 0.42; I^2^*:* 56%). The time that children spend at school is less than the time they spend outside of it. A comprehensive model that considers different parts of the day might be more helpful in reducing SB throughout the day. A fundamental axis may be active interventions into school hours, which have been shown to be effective in increasing PA and MVPA throughout the day. In addition, these interventions should not only focus on the child, but also on the families; they should commit to raising awareness about the harming effects of a sedentary lifestyle, especially during childhood [13].

Analyzing methodological quality and risk of bias, it was observed that most of the included studies showed the same biases. In all studies, blinding the participants was not possible, as well as the teachers in charge of performing the intervention [19,20,21,22,23,24]. In a school-based study environment, performing this type of blinding is complicated, but at the same time, this fact causes a bias. Another detected risk of bias was related to the lack of a proper registration of adherence, causing either high losses of follow-up or making it difficult to assure the total dosage received, or, in this case, performed by children [19,21,22]. In addition, the inclusion criteria of the subjects were not reported in the included studies [19,20,21,22,23,24], although some of them showed the description of the sample characteristics in the methodology section. This fact has been positively considered when analyzing the methodological quality of the studies with the PEDro scale, as there is no intermediate punctuation. A difference in sample sizes of control and experimental groups from the beginning of the studies [19,21,24] has been detected. In line with the latter commented, we found some discrepancies in the baseline information, with only the studies of Drummy et al. [20] and Masini et al. [22] showing baseline data for the outcome measures of interest in this review.

Some limitations were identified in the present study. Differences in the intervention to interrupt sitting time could have added a source of error to the pooled results. There is a large heterogeneity among the included studies in terms of duration of sitting time interruption, frequency and total program duration. The tools used to measure PA, MVPA and SB were not homogeneous across studies; the meta-analyses included studies using accelerometers [19,20,22] and one study [21] using activity trackers. Differences in activity data collection could be another source of error in the results presented in this review. 

## 5. Conclusions

It seems that active school-based interventions to interrupt prolonged sitting are effective at improving daily physical activity and moderate-to-vigorous physical activity, but the intervention is not enough to reduce daily sedentary behavior. More research with homogeneous interventions, with higher methodological quality and less bias, is needed to determine the real effects of active school-based interventions in the medium and long term. WHO recommendations should be considered to develop new intervention protocols, focusing on the sedentary behavior and not only on physical activity programs.

## Figures and Tables

**Table 1:** Characteristics of the included studies.

Reference	Year	Country	Age		N	Sitting Time Interruption	Outcome Measures				
			Age Range	Mean Age (SD)			PA	MVPA	SB	Tool	Other Outcome Measures
Donelly, 2009 [19]	2009	USA	6–9	2nd FEG 7.7 (0.3) 2nd FCG 7.8 (0.4) 2nd MEG 7.7 (0.4) 2nd MCG 7.8 (0.3) 3th FEG 8.7 (0.4) 3th FCG 8.7 (0.4) 3th MED 8.7 (0.3) 3th MCD 8.8 (0.4)	454	Weekly 90 min (10-min bout) for 3 years	x	x		Accelerometer (ActiGraph, 7163, Pensacola, FL)	BMI and academic achievement
Drummy, 2016 [20]	2016	Ireland	9–10	9.5	107	5 min 3 times/day for 12 weeks		x		Accelerometer (Actigraph GT1M, Actigraph LLC, Pensacole, FL, USA)	BMI and skinfold measurements
Buchele, 2018 [21]	2018	USA	10–11	x	116	Daily 6 min for 4 weeks (PAEB-C program)	x	x	x	Activity tracker (Fitbit Charger + Heart RateTM)	aerobic fitness
Masini, 2020 [22]	2020	Italy	8–9	9.02 (0.11)	28	10 min twice a day for 14 weeks	x	x	x	Accelerometer (ActiGraph wGT3X-BT, Actigraph LLC, Pensacole, FL, USA)	satisfaction
Pinto-Escalona, 2019 [23]	2019	Spain	13–14	13.6 (0.7)	116	Daily 10 min for 5 days	x			Questionnaire for Assessing Physical Activity in Teenage Schoolchildren	academic achievement and attention
Reed, 2010 [24]	2010	USA	8–9	EG 9.42 CG 9.50	155	30 min 3 times/week for 4 months	x			Questionnaire Previous Day Physical Activity Recall (PDPAR)	fluid intelligence and academic achievement

FEG: female experimental group, FCG: female control group, MEG: male experimental group, MCG: male control group, EG: experimental group, CG: control group, BMI: body mass index.

**Table 2:** PEDro scale scores.

		1. Eligibility Criteria Were Specified	2. Random Allocation	3. Concealed Allocation	4. Groups Similar at Baseline	5. Participants Blinding	6. Teachers Blinding	7. Outcome Assessors Blinding	8. Less than 15% Dropouts	9. Intention to Treat Analysis	10. Between-Group Statistical Comparisons	11. Point Measures and Variability Data	TOTAL
[19]	Donnelly, 2009	1	1	0	1	0	0	1	1	0	1	1	6
[20]	Drummy, 2016	1	1	0	1	0	0	0	1	0	1	1	5
[21]	Buchele, 2018	1	0	0	0	0	0	0	1	0	1	1	3
[22]	Masini, 2020	1	0	0	0	0	0	0	0	0	1	1	2
[23]	Pinto-Escalona, 2019	1	1	0	1	0	0	0	1	0	1	1	5
[24]	Reed, 2010	1	1	0	1	0	0	0	0	0	1	1	4
	TOTAL	6	4	0	4	0	0	1	4	0	6	6	4.2

## Data Availability

Not applicable.

## Supplementary Materials

The following supporting information can be downloaded at: https://www.mdpi.com/article/10.3390/ijerph192215409/s1, Supplementary material: Search Strategy.
